# Cost-effectiveness analysis of personalised versus standard dosimetry for selective internal radiation therapy with TheraSphere in patients with hepatocellular carcinoma

**DOI:** 10.3389/fonc.2022.920073

**Published:** 2022-08-29

**Authors:** Carla Rognoni, Maria Rosa Barcellona, Irene Bargellini, Maria Grazia Bavetta, Marilena Bellò, Maurizia Brunetto, Patrizia Carucci, Roberto Cioni, Laura Crocetti, Fabio D’Amato, Mario D’Amico, Simona Deagostini, Désirée Deandreis, Paolo De Simone, Andrea Doriguzzi, Monica Finessi, Paolo Fonio, Serena Grimaldi, Salvatore Ialuna, Fabio Lagattuta, Gianluca Masi, Antonio Moreci, Daniele Scalisi, Roberto Virdone, Rosanna Tarricone

**Affiliations:** ^1^ Centre for Research on Health and Social Care Management (CERGAS), SDA Bocconi School of Management, Bocconi University, Milan, Italy; ^2^ Azienda Ospedaliera Ospedali Riuniti Villa Sofia Cervello, Palermo, Italy; ^3^ Azienda Ospedaliero Universitaria Pisana, Pisa, Italy; ^4^ Azienda Ospedaliero Universitaria Città della Salute e della Scienza, Torino, Italy; ^5^ Department of Policy Analysis and Public Management, Bocconi University, Milan, Italy

**Keywords:** trans-arterial radioembolisation, cost-effectiveness, cost-utility, personalised dosimetry, tailored treatment

## Abstract

**Aims:**

To perform a cost-effectiveness analysis (CEA) comparing personalised dosimetry with standard dosimetry in the context of selective internal radiation therapy (SIRT) with TheraSphere for the management of adult patients with locally advanced hepatocellular carcinoma (HCC) from the Italian Healthcare Service perspective.

**Materials and methods:**

A partition survival model was developed to project costs and the quality-adjusted life years (QALYs) over a lifetime horizon. Clinical inputs were retrieved from a published randomised controlled trial. Health resource utilisation inputs were extracted from the questionnaires administered to clinicians in three oncology centres in Italy, respectively. Cost parameters were based on Italian official tariffs.

**Results:**

Over a lifetime horizon, the model estimated the average QALYs of 1.292 and 0.578, respectively, for patients undergoing personalised and standard dosimetry approaches. The estimated mean costs per patient were €23,487 and €19,877, respectively. The incremental cost-utility ratio (ICUR) of personalised versus standard dosimetry approaches was €5,056/QALY.

**Conclusions:**

Personalised dosimetry may be considered a cost-effective option compared to standard dosimetry for patients undergoing SIRT for HCC in Italy. These findings provide evidence for clinicians and payers on the value of personalised dosimetry as a treatment option for patients with HCC.

## Introduction

Globally, there are about 840,000 new cases of liver cancer each year (1). Primary liver cancer is the second leading cause of cancer death worldwide (2). Seventy-five percent of liver cancers are hepatocellular carcinoma (HCC) resulting from cirrhosis. Patients are typically diagnosed late in the disease, with a relatively small percentage eligible for curative treatments. Despite the addition of several new therapies for advanced HCC, the 5-year survival rate is 18% (3). Interventional techniques, like radiation-based approaches, are of interest because they are known to be cytocidal in adequate doses and are independent of other chemical or energy-based treatment techniques. While a delivery of more than 70 Gray (Gy) is considered necessary to achieve necrosis of a solid tumour (4), the tolerance of healthy liver tissue is about 30 Gy (5). These conditions were the basis of the development of liver-directed selective internal radiation therapy (SIRT) or brachytherapy, also referred to as transarterial radioembolisation (TARE) therapy. SIRT or TARE requires an infusion of radioactive microspheres, loaded with yttrium-90 (Y90) or, more recently, holmium-166 (Ho166).

In this context, TheraSphere is approved for the treatment of hepatic malignancies in Europe (CE mark indication). TheraSphere is made up of glass microspheres in which the Y90 radioactive isotope is imbedded. These microspheres are infused by an interventional radiologist and nuclear medicine physicians into the hepatic artery *via* a catheter and become lodged in capillaries within the tumour vasculature. With a penetration range of 2.5 mm, the emitted radiation destroys the cancer cells over a period of approximately 2 weeks. The aim of the treatment, with the standard dosimetric approach, is to deliver an absorbed dose of 120 ± 20 Gy to the treated hepatic volume.

Recent retrospective studies have shown that a personalised and optimised dosimetric approach, which takes into account the dose absorbed by the tumour, is technically possible and could lead to higher response rates (6, 7). The recently published DOSISPHERE-01 trial (8) is a randomised, multicentre, open-label phase 2 trial done at four healthcare centres in France with the aim to compare personalised to standard dosimetry. Dosimetry was evaluated by medical physicists and nuclear medicine physicians using a local software [volumetric analysis (Syngo Workstation, Siemens, Malvern, PA, USA) and PLANET Dose (DOSIsoft, Paris, France)]. The target in the personalised dosimetry approach was to deliver at least 205 Gy to the tumour, arriving at more than 250 Gy, if possible. The results of the study showed that, compared with standard dosimetry, personalised dosimetry significantly improved the overall survival and the objective response rate in patients with locally advanced hepatocellular carcinoma. Moreover, personalised dosimetry is likely to improve patient outcomes in clinical practice.

The aim of this study is to support stakeholders in the evaluation of the treatment choices in patients with HCC. Specifically, this study evaluated the cost-effectiveness of personalised dosimetry to standard dosimetry in the context of SIRT with TheraSphere from the National Healthcare Service (NHS) perspective in Italy.

## Materials and methods

Medical devices have distinctive features compared to drugs, such as incremental innovation, dynamic pricing, a learning curve, and organisational impact (e.g., the need to create adequate operating rooms), that need consideration when evaluated (9). The study followed the Consolidated Health Economic Evaluation Reporting Standards (CHEERS) reporting guideline (10), supplemented by the distinct features of medical devices (11). The checklist is highlighted in **Appendix 1**.

### The model

A partition survival model with “stable disease,” “progression,” and “death” health states has been developed to estimate life years (LYs), quality-adjusted life years (QALYs), and the costs associated with personalised dosimetry and standard dosimetry in the context of SIRT with TheraSphere. The model considers the same population analysed by Garin and colleagues (8): adult population with a mean age of 64 years (91.5% men) with locally advanced hepatocellular carcinoma not amenable to surgery or local ablative treatment, BCLC (Barcelona Clinic Liver Cancer) B (intermediate stage) and C (advanced stage) (mainly C). This population reflects the indications of national (12) (Italy) and international guidelines (13, 14) for the management of hepatocellular carcinomas through SIRT.

**Figure 1:**
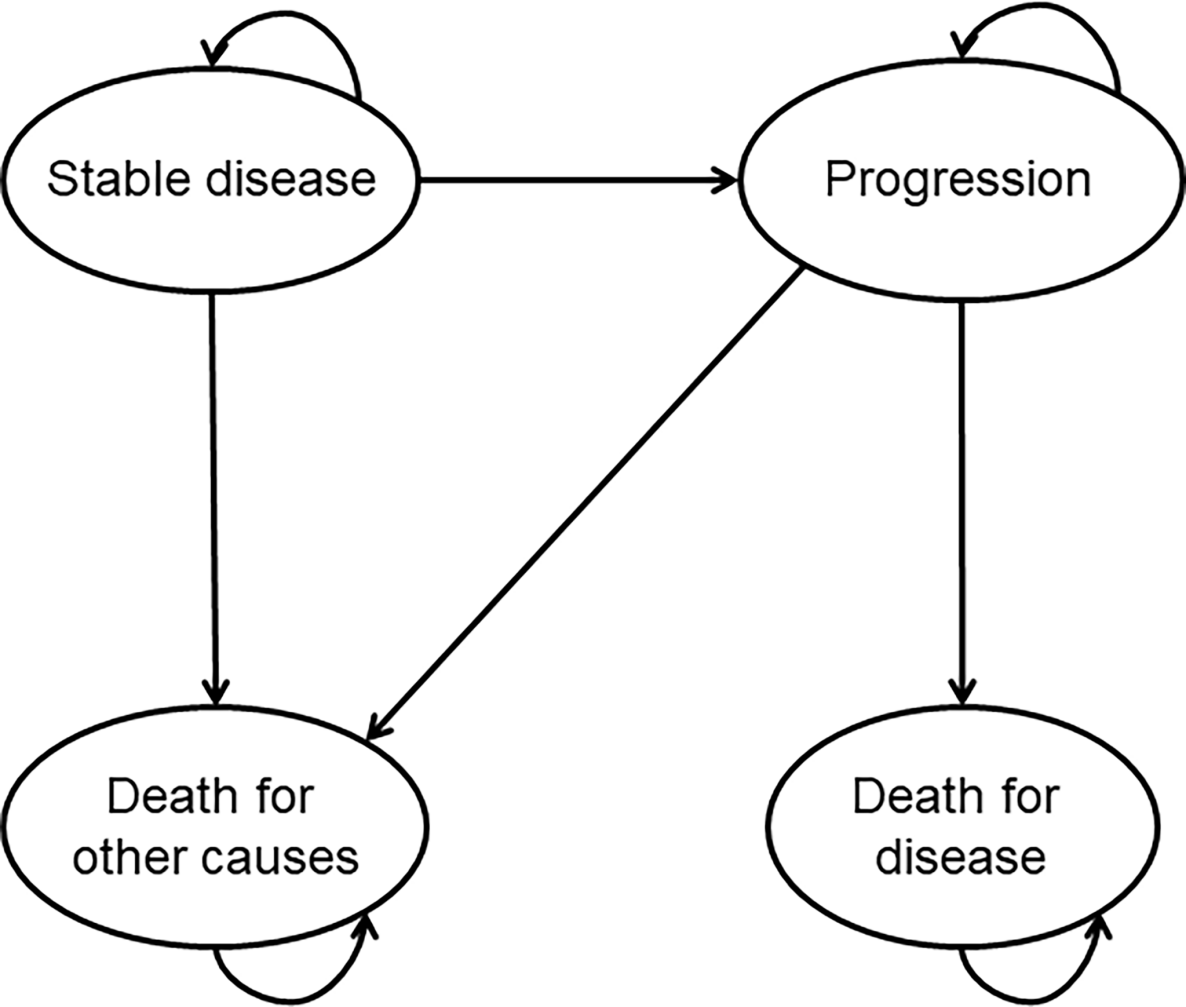
Representation of the implemented model.

All patients start in the “stable disease” health state. In cases of disease progression, patients move to the “progression” state. Once patients have progressed their disease, they cannot return to their previous health status. Once patients transition into the state of “death,” they remain in that state until the end of the process (**Figure 1**). The model considers a cycle length of 1 month and applies a “half-cycle” correction for costs and benefits. The partition survival model, which is now used in a significant proportion of appraisals (15, 16), may be seen as a relatively straightforward and intuitive approach because state occupancy can be estimated directly from trial-based estimates of survival. Indeed, patients’ distribution over time among the different health states has been derived from overall survival (OS) and progression-free survival (PFS) data available from the randomised controlled trial (RCT) DOSISPHERE-01 by Garin and colleagues (8). The model applies a time horizon of 45 years; given the median age of 64 years of the population considered, 45 years was considered long enough to cover the lifetime of every patient. In order to estimate clinical and economic outcomes over a lifetime horizon, clinical trial data were extrapolated through curve fittings. For standard dosimetry, for each clinical outcome (OS, PFS), the parameters were “fitted” with different functions, and the most plausible models were selected by statistical methods (see **Appendix 2** for details). Specific hazard ratios (personalised dosimetry vs. standard dosimetry, HR_OS_ = 0.421, HR_PFS_ = 0.71) reported in (8) were applied to both OS and PFS curves of standard dosimetry to obtain the curves for personalised treatment.

Mortality rates were further adjusted for age and gender according to the Italian mortality tables (ISTAT) to consider deaths caused by other comorbidities (“Death for other causes” state). A discount rate of 3% was applied to health outcomes and costs (17).

### Healthcare resource utilisation and costs

**Table 1** reports the unit cost of the resources used for treatment with TheraSphere with personalised or standard dosimetry (€, 2021). The frequency of use was elicited through the administration of a questionnaire to clinicians from departments (oncology, interventional radiology, nuclear medicine, physics, and gastroenterology) in three clinical centres that are geographically distributed in Italy and perform a high volume of SIRT procedures annually (Azienda Ospedaliero Universitaria Pisana, Pisa; Azienda Ospedaliera Ospedali Riuniti Villa Sofia Cervello, Palermo; Azienda Ospedaliero Universitaria Città della Salute e della Scienza, Torino). Clinicians in each clinical centre completed the questionnaire in a collaborative way reporting data according to their clinical practice (12). The mean value for healthcare resource utilisation was calculated from the completed questionnaires. The model considers different healthcare services that are used in the peri-procedural period (in the first month, exams/visits performed before and after SIRT), together with other healthcare services used in the follow-up (stable disease or progression).

**Table 1:** Input parameters: healthcare resource utilisation and related unit costs.

Parameter	Base case value	Frequency in the peri-procedural period	Frequency in stable disease	Frequency in progression	Reference
Specialist visit	€20.66	1.00	0.33	1.00	National tariff
Full blood counts	€3.17	1.00	0.33	1.00	National tariff
Creatinine	€1.13	1.00	0.33	1.00	National tariff
Sodium	€1.02	1.00	0.33	1.00	National tariff
Potassium	€1.02	1.00	0.33	1.00	National tariff
Calcium	€1.13	0.50	0.17	1.00	National tariff
Prothrombin time	€2.85	1.00	0.33	1.00	National tariff
Albumin	€1.42	1.00	0.33	1.00	National tariff
Bilirubin	€1.13	1.00	0.33	1.00	National tariff
Alpha-fetoprotein	€7.40	1.00	0.33	1.00	National tariff
Aspartate aminotransferase (GOT)	€1.04	0.33	0.11	–	National tariff
Alanine aminotransferase	€1.00	1.00	0.33	1.00	National tariff
Gamma-glutamyl transpeptidase (GPT)	€1.13	0.83	0.28	1.00	National tariff
Alkaline phosphatase	€1.04	1.00	0.33	0.67	National tariff
Pseudocholinesterase (PCHE)	€1.36	0.33	0.11	–	National tariff
CT scan (abdomen)	€103.68	0.80	0.27	0.16	National tariff
CT scan (thorax)	€77.67	0.33	0.11	0.11	National tariff
RMN abdomen	€120.08	0.20	0.07	0.03	National tariff
PET post-treatment	€1,071.65	0.67	–	–	National tariff
SPECT post-simulation	€25.93	1.00	–	–	National tariff
Ultrasound abdomen	€60.43	0.17	0.06	0.17	National tariff
Gastroscopy	€56.81	0.08	0.03	0.01	National tariff
Paracentesis	€34.86	–	–	0.15	National tariff

Concerning SIRT treatment, a simulation and the treatment were considered for each patient. The simulation was used to evaluate the anatomy of the arterial supply to the liver, optimise the condition of the arteries to convey the treatment, and avoid side effects. The simulation referred to the diagnosis-related group (DRG) 203 (€4,085, national tariff), while the treatment referred to different reimbursement rates in the different Italian regions by their specific tariff and/or extra tariff DRGs (DRG 203 “Malignancy of hepatobiliary system or pancreas,” DRG 191 “Pancreas, liver & shunt procedures w cc,” DRG 192 “Pancreas, liver & shunt procedures w/o cc,” DRG 409 “Radiotherapy”). As such, reimbursement ranged between €1,279.77 and €22,364.35 (patients with comorbidities). In this analysis, we used the mean reimbursement rate from the four DRGs (national tariff), being the same reimbursement value considered for both strategies (SIRT with personalised or standard dosimetry). This meant that the choice of the tariff did not influence the results from the cost-effectiveness analysis (CEA). For personalised dosimetry, a cost of €150 for each SIRT procedure was applied for the software license. Based on the self-reported data collected from the questionnaires, the model accounting for 13% of the HCC cases required a coil embolisation to perform SIRT (DRG 203, national tariff €4,085).

Data collected from clinicians were used to detail the pharmacological treatments administered to patients following the procedure or in the progression phase. The former was mainly composed of antibiotic and anti-inflammatory therapy (3.5 g of ceftriaxone, 42% of patients; 875 cc of physiologic solution, 42% of patients; betamethasone 40 mg, 33% of patients; ursodeoxycholic acid 300 mg/day for 3 months, 33% of patients; potassium perchlorate 400 mg, 33% of patients) for a mean cost per patient of €11.20. The latter consisted of antibiotics (10 g of ceftriaxone, 24 g of piperacillin/tazobactam, 2% of patients), diuretics (75 mg of furosemide, 300 mg of potassium canrenoate, 2% of patients), and albumin (375 ml, 2% of patients) for a mean cost of €3.88 per patient.

Subsequent treatments were identified from the available literature (8) and incorporated into the model. They were liver resections (DRG 192, national tariff €9,558) performed in 36% and 4% of patients for personalised and standard dosimetry, respectively. It was assumed that subsequent treatments were performed after objective response evaluation (3 months after the SIRT) (8).

Current guidelines do not report recommendations about the specific case of progression management after SIRT treatment. In general, a systemic treatment may be performed in patients with advanced stage of disease (13, 14). Recently, lenvatinib showed non-inferiority efficacy compared with sorafenib, which was the established standard systemic therapy for HCC according to all international guidelines following the results reported a decade ago (18). We therefore conducted a scenario analysis by considering the cost of treatment with lenvatinib for patients progressing after SIRT. According to the data presented in (18), we considered a mean dose per day of 9.47 mg and a median duration of treatment of 5.7 months. Regarding the cost of lenvatinib, we referred to published Italian official tariffs (cost of €14.10 per mg) (19).

For terminal care, the model considered the cost of best supportive care reported in the literature for the Italian setting (€4,142—year 2012 corresponding to €4,374—year 2021) (20).

### Quality-of-life estimates

Utility coefficients (0.51 for stable disease and 0.35 for progression) related to the health states were obtained from a published study (21), which estimated them from an analysis of studies in the Cost-Effectiveness Analysis Registry (22). Since the utility values of health states are not specific to the dosimetric approach used, we applied disutilities related to adverse events to capture the difference in the quality of life (QOL) between the two treatment options. Disutilities were retrieved from the cited registry. Since more than one study reported disutilities data, a mean value was estimated which took into account the highest quality degree [studies were classified on a quality scale from 1 (low) to 7 (high)]. For the duration of the adverse events, the threshold values in days for the different DRGs were considered (according to Italian Ministerial Decree 18/12/2008). **Table 2** shows a summary of data related to the modelling of adverse events.

**Table 2:** Disutilities, frequencies, durations, and costs of adverse events used in the model.

Adverse event	Disutility		Management			% of patients		
	Value	Ref.	Mean duration (days)	Tariff	Ref.	Personalised dosimetry	Standard dosimetry	Ref.
Lymphopenia	0.09	(23)	17	€1,704	DRG 399	34%	43%	(8)
Asthenia	0.12	(24–26)	17	€1,745	DRG 247	–	5%	(8)
Ascites	0.21	(27)	23	€1,748	DRG 464	–	10%	(8)
Increased blood bilirubin	0.06	(28)	21	€1,407	DRG 206	–	5%	(8)
Increased aspartate aminotransferase	0.06	(28)	21	€1,407	DRG 206	9%	10%	(8)
Anaemia	0.11	(24–26, 29–34)	23	€1,676	DRG 395	6%	5%	(8)
Thrombocytopenia	0.23	(23, 25, 29, 32, 33)	20	€2,748	DRG 397	–	5%	(8)
Decreased weight	0.03	(35)	21	€1,758	DRG 297	–	5%	(8)
Increased alanine aminotransferase	0.06	(28)	21	€1,407	DRG 206	9%	–	(8)
Gastrointestinal haemorrhage	0.11	(36)	13	€959	DRG 183	–	10%	(8)
Icterus	0.30	(37)	21	€1,407	DRG 206	–	10%	(8)

### Cost-effectiveness analysis

The incremental cost-effectiveness ratio (ICER) was calculated as the difference in the mean expected costs (i.e., incremental cost, Δ*C*) between personalised and standard dosimetry divided by the difference in the mean expected outcomes (i.e., incremental life years, Δ*E*) between these strategies (ICER = Δ*C*/Δ*E*). We referred to the incremental cost-utility ratio (ICUR) when effectiveness is expressed in QALYs.

As the long-term curve extrapolations may influence the results of the analyses, shorter horizons of 5 and 10 years have been also considered.

Deterministic and probabilistic sensitivity analyses (PSA) were performed to test the robustness of the model. The PSA was performed by assigning distributions to model parameters (beta for utilities, log-normal for hazard ratios, gamma for costs, and frequencies of events). In case the studies referencing the parameters reported 95% confidence intervals, these were applied to estimate parameter variations; otherwise, a standard deviation of 20% of the baseline value was used. For the PSA, 1,000 Monte Carlo simulations were performed by randomly sampling all the parameters from their assigned distributions. Results have been presented graphically as scatterplots in the cost-effectiveness plane. Results of the univariate analysis are reported as a tornado diagram for the ICUR. **Supplementary Table 1** shows the details regarding the parameters used in the analyses.

## Results

### Cost-effectiveness analysis

Considering a lifetime horizon, the model estimated the average QALYs of 1.292 and 0.578, respectively, for patients undergoing personalised and standard dosimetry approaches. The estimated mean costs per patient were €23,487 and €19,877 for personalised and standard options, respectively. The model results for the two scenarios are summarised in **Table 3**. The ICUR of personalised versus standard dosimetry approaches was €5,056/QALY, highlighting the cost-effectiveness of the tailored procedure (ICUR < €50,000). The cost-effectiveness of personalised versus standard dosimetry was confirmed also considering the shorter time horizons of 5 and 10 years for the analyses (**Table 3**).

**Table 3:** Model results according to different time horizons.

Expected outcomes	Standard dosimetry	Personalised dosimetry	Difference	ICER or ICUR
Lifetime horizon				
Costs	€19,877	€23,487	€3,609	
LYs	1.358	3.226	1.868	€1,932
QALYs	0.578	1.292	0.714	€5,056
Time horizon = 5 years				
Costs	€19,824	€22,420	€2,596	
LYs	1.304	2.367	1.063	€2,443
QALYs	0.557	0.981	0.423	€6,131
Time horizon = 10 years				
Costs	€19,870	€23,164	€3,294	
LYs	1.351	2.918	1.568	€2,101
QALYs	0.575	1.182	0.607	€5,429

**Table 4** shows the detailed costs for each treatment strategy and by health states considered in the model. Personalised treatment, compared to standard dosimetry, led to lower costs for the management of adverse events and higher costs for second-line treatments like liver resections. The difference in subsequent treatment cost was due to the higher effectiveness of personalised dosimetry in improving patients’ outcomes, allowing patients to undergo curative liver resection. Terminal care costs are lower for the personalised dosimetry approach due to the lower number of patients dying from the disease.

**Table 4:** Cost details.

Model health state	Healthcare resources	Cost of healthcare resources		Total cost for model health state	
		Standard dosimetry	Personalised dosimetry	Standard dosimetry	Personalised dosimetry
Stable disease	Radioembolisation	€11,847	€11,997	€14,947	€14,586
	Pharmacological treatments	€11	€11		
	Management of AEs	€1,696	€919		
	Visits/exams	€1,393	€1,659		
Progression	Visits/exams	€715	€2,329	€1,098	€5,747
	Subsequent treatments	€383	€3,419		
Death	Terminal care	€3,832	€3,154	€3,832	€3,154

The scenario analysis considering the cost of systemic chemotherapy for progressive patients led to mean costs per patient of €44,078 and €41,292 for personalised and standard dosimetry, respectively, showing a lower ICUR (€3,903/QALY) compared to the base case analysis.

**Figure 2:**
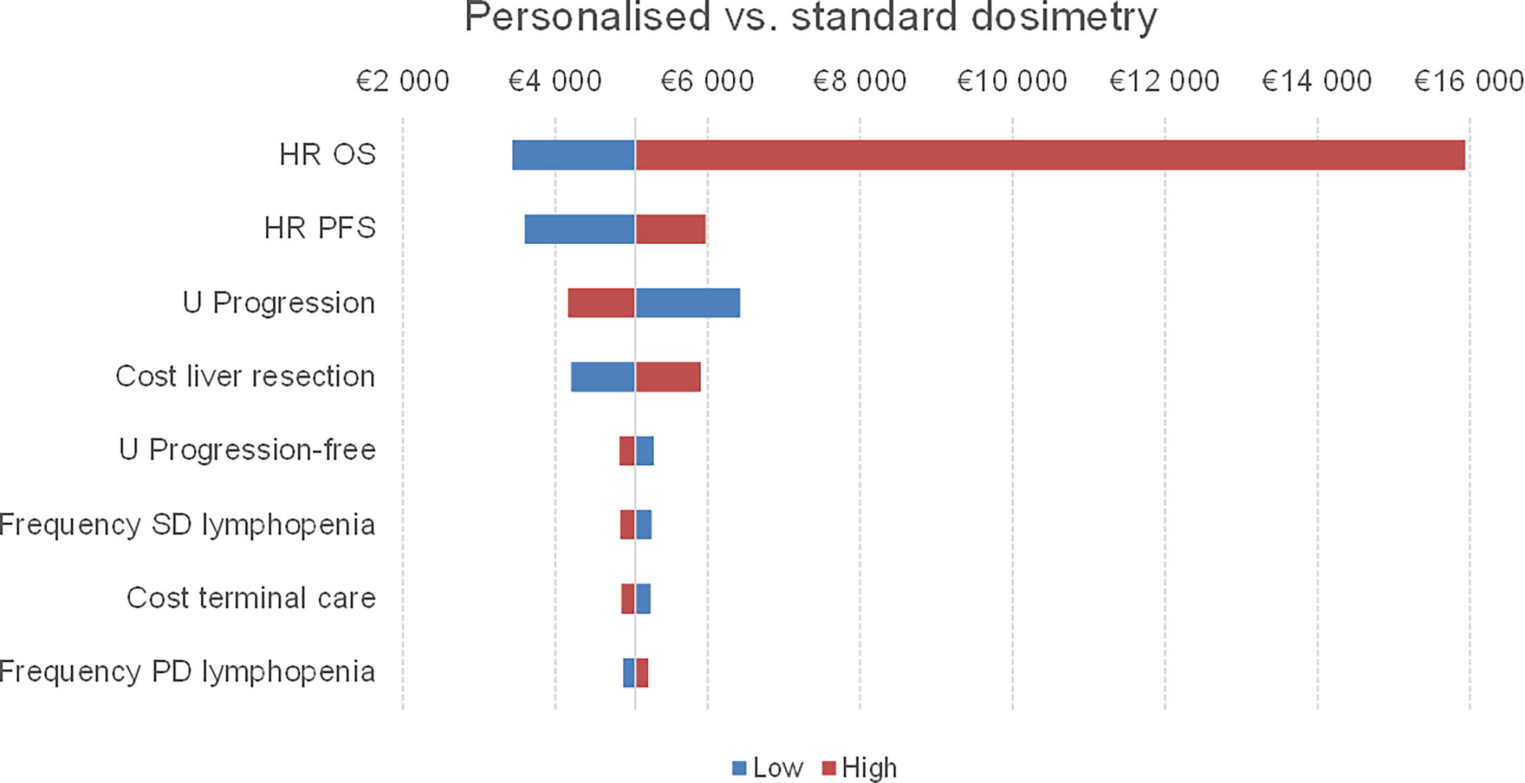
Tornado diagram of one-way sensitivity analysis of personalised and standard dosimetry for the ICUR. The vertical line represents the incremental value between the two strategies using the base case value for each parameter. As the parameters deviate from their base case values, the ICUR changes. The red bar represents the variation of the ICUR when the parameter ranges from the base case to the high uncertainty value. Conversely, the blue bar shows the ICUR variation when the parameter ranges from the low uncertainty value to the base case value. SD, standard dosimetry; PD, personalised dosimetry.

Tornado diagrams reporting a one-way sensitivity analysis for the ICUR are reported in **Figure 2**. The most impactful parameters reporting greater variations on the ICUR were the hazard ratios for OS and PFS, the utility value for progression, and the cost for liver resection as subsequent treatment.

**Figure 3:**
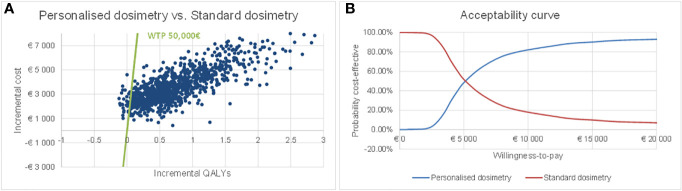
Probabilistic sensitivity analysis results: scatterplot in the cost-effectiveness plane **(A)** and acceptability curve **(B)**.

Concerning the PSA, the plot of incremental costs versus incremental QALYs obtained from the Monte Carlo simulations is shown in **Figure 3A**. The dotted line represents a theoretical cost-effectiveness willingness-to-pay threshold of €50,000/QALY. Nearly all the simulations (96.7%) were below this line. **Figure 3B** reports the acceptability curve.

## Discussion

The first approach to medicine was “disease-based,” meaning that patients were treated only after disease manifestation. In this case, the diagnosis was mainly based on the signs and symptoms, and treatment was prescribed according to the experience of the physician. The current approach is described as “evidence-based” because the diagnostic–therapeutic pathway is conducted according to the outcome of clinical studies and clinical practice guidelines, developed based on clinical research. With this approach, it is possible to know the mechanisms of diseases and to implement the most appropriate therapy. The newer “personalised” approach tailors medical treatment to the individual characteristics of each patient (e.g., lifestyle parameters, genome, microbiome). As such, the ability to predict which medical treatments will be safe and effective for each patient is improved and the costs are contained (38). In this context, personalised dosimetry for SIRT with TheraSphere in patients with HCC improved the treatment workflow leading to better patient outcomes (8). SIRT with personalised dosimetry allowed for the improvement of patient outcomes enough to consent to curative resection surgery of the liver (liver resection was possible in 36% of the patients that previously had SIRT with personalised dosimetry compared to 4% of the patients who underwent SIRT with standard dosimetry). Moreover, the best clinical outcome of personalised dosimetry (i.e., improved overall survival) is obtained with a limited increase in costs (€150) for the procedure preparation. Regarding this aspect, dosimetry could also be performed without any commercial software but through the imaging workstation with the use of a datasheet, and this setting would allow to remove also this low additional fee (39).

Several studies comparing treatments for HCC exist, but to our knowledge, this study is the first to evaluate the cost-effectiveness of different types of dosimetry modalities. Although in Italy there is no official threshold to be considered in cost-effectiveness studies, the Italian Association of Health Economics suggests a threshold between €25,000 and €40,000 per QALY (40), while a published study (41) defined a cost-effective treatment whose maximum value per month of life gained is less than €5,000 (i.e., €60,000 per year gained). Considering an intermediate willingness-to-pay threshold of €50,000, this study showed that TheraSphere (SIRT) with personalised dosimetry for the treatment of patients with HCC may be a cost-effective option in comparison to the standard dosimetry approach. The clinical effectiveness of personalised and standard dosimetry was derived from a recently published RCT. The results from the RCT were robust and evaluated through one-way and probabilistic sensitivity analyses. The results showed that personalised dosimetry reduced the burden of adverse events, which is a non-negligible aspect that may affect from 4% to 17% of patients (42–46).

SIRT may be performed with different types of microspheres such as glass or resin. The ideal radiation therapy is to deliver lethal doses of radiation as high as possible to the tumour while protecting the surrounding normal liver parenchyma. In this analysis, we referred to glass microspheres as they were considered in the reference study (8). Preliminary data, investigating the dose of radiation delivered through glass versus resin-based Y90 SIRT in patients with intrahepatic cholangiocarcinoma, showed a significantly higher ratio of Y90 dose delivered to the tumour versus normal liver in the glass group compared to the resin group (4.9 ± 0.7 vs. 2.4 ± 0.3, respectively, p < 0.001) (47). The conclusions of this analyses are valid for glass microspheres, and we recommend that future studies should replicate this research to other types of microspheres.

Our study has some limitations. First, medical devices show particular challenges for health technology assessments caused by rapid innovation, outcomes influenced by training, the competence of the final users, and dynamic pricing (9). The reference study (8) did not report data on the technical success of the SIRT procedure. In patients with complications, the management cost may increase, potentially compromising the results of this study. This concept is related also to the experience of the operators who perform the interventions. It has been demonstrated that clinical outcomes and resource consumption related to patients managed with new technologies, such as SIRT, may be strongly influenced by the underlying learning curve of the operators (11). Also, centres performing a higher volume of procedures may obtain better performance of the device, yield better health outcomes, and lower procedure costs (11). Continuous data collection and monitoring could provide more robust data for the evaluation of these aspects.

Second, the model was populated according to clinical outcomes reported in a single RCT with a limited number of enrolled patients, and the generalisability of the model results to a broader real-world population, for example to patients with small lesions (i.e., <3 cm), should be performed with caution. Another point relates to the impact of disease severity or risk factors on the cost-effectiveness of personalised dosimetry. The good safety profile of the tailored approach is probably the result of accurate patient selection, with the inclusion of patients with good liver function and a hepatic reserve of at least 30% after selective internal radiation therapy (8); on the other side, baseline liver function abnormalities, prior to radioembolisation, have shown to be predictors of post-treatment toxicities (48). Also, the amount of activity administered to target the liver volume can be considered a risk factor of experiencing SIRT-related side effects (49). In this context, it is likely that patients treated in a real-world setting with personalised dosimetry experience the worst side effects, thus resulting in lower quality of life and higher management costs, leading to a possible worsening of the cost-effectiveness profile versus standard dosimetry.

Third, our model did not include systemic treatments (chemotherapy for progressed patients) performed after SIRT but focused on treatments like liver resections. Nevertheless, these results may be considered conservative because costs are likely to be higher for standard dosimetry, with a higher progression rate compared to personalised treatment. This result was also confirmed by the scenario analysis conducted considering treatment with systemic chemotherapy (lenvatinib) for progressive patients. With the limited availability of RCT data, comparative observational studies, patient registries, or claims databases may be suitable options for the generation of real-world evidence on the effectiveness and safety of medical devices to support health technology assessments (50–52).

Fourth, this model did not take into consideration the organisational impact of the constitution of the interventional radiology and nuclear medicine unit together with a medical physicist in a clinical centre (11, 53). However, it may be used for different purposes, as it entails substantial investments (e.g., adequate spaces, equipment, dedicated personnel, creation of multidisciplinary teams) to implement the diagnostic–therapeutic pathway for the treatment of patients with radiation therapy. Although the investment is initial and this aspect should not influence the comparison between the two treatment options considered, for personalised dosimetry, the cost of a training plan for nuclear physicians and medical physicists may be envisaged. Finally, data on patients’ QOL were derived from published literature, and the transferability of the retrieved utility values to an Italian context was not considered.

Our findings showed that personalised dosimetry for the management of SIRT in patients with HCC may be a cost-effective choice compared to standard dosimetry. These findings provide evidence for clinicians and payers on the value of personalised dosimetry as a treatment option for patients with HCC. An innovative treatment option is now available to clinicians who may offer an appropriate and customised management plan to improve clinical outcomes in patients. For now, decision-makers may use these preliminary results to support a tailored approach in defining and treating the targeted patient populations. Future studies comparing the personalised approach to the standard approach are recommended to increase the clinical evidence to confirm or reject the validity of this preliminary evaluation.

## Data availability statement

The original contributions presented in the study are included in the article/**Supplementary Material**. Further inquiries can be directed to the corresponding author.

## Author contributions

CR and RT contributed to the conception and design of the study. CR performed the cost-effectiveness analysis. CR wrote the first draft of the manuscript. RT supervised the manuscript preparation. MRB, IB, MGB, MBe, MBr, PC, RC, LC, FD’A, MD’A, SD, DD, PS, AD, MF, PF, SG, SI, FL, GM, AM, DS, and RV provided data on healthcare resource consumption. All authors contributed to manuscript revision and read and approved the submitted version.

## Funding

This study was funded by Confindustria Dispositivi Medici Servizi Srl through an unrestricted grant to CERGAS, SDA Bocconi School of Management, *via* Sarfatti 10, 20136 Milan, Italy.

## Conflict of interest

The authors declare that this study received funding from Confindustria Dispositivi Medici Servizi Srl. The funder was not involved in the study design, collection, analysis, interpretation of data, the writing of this article or the decision to submit it for publication.

## Publisher’s note

All claims expressed in this article are solely those of the authors and do not necessarily represent those of their affiliated organizations, or those of the publisher, the editors and the reviewers. Any product that may be evaluated in this article, or claim that may be made by its manufacturer, is not guaranteed or endorsed by the publisher.
