# Supplementary material for: Cost-effectiveness analysis of personalised versus standard dosimetry for selective internal radiation therapy with TheraSphere in patients with hepatocellular carcinoma
Source: Front Oncol. 2022 Aug 29;12:920073. doi: 10.3389/fonc.2022.920073 (PMC9464985; doi:10.3389/fonc.2022.920073)
Supplement: Supplementary file 1 [file DataSheet_1.docx]

**Appendix 1**

**CHEERS checklist—Items to include when reporting economic evaluations of health interventions**

| **Section/item** | **Item No** | **Definition** | **Reported/not reported** |
| --- | --- | --- | --- |
| **Title and abstract** | | | |
| Title | 1 | Identify the study as an economic evaluation or use more specific terms such as “cost-effectiveness analysis”, and describe the interventions compared. | Reported |
| Abstract | 2 | Provide a structured summary of objectives, perspective, setting, methods (including study design and inputs), results (including base case and uncertainty analyses), and conclusions. | Reported |
| **Introduction** | | | |
| Background and objectives | 3 | Provide an explicit statement of the broader context for the study.  Present the study question and its relevance for health policy or practice decisions. | Reported |
| **Methods** | | | |
| Target population and subgroups | 4 | Describe characteristics of the base case population and subgroups analysed, including why they were chosen. | Reported |
| Setting and location | 5 | State relevant aspects of the system(s) in which the decision(s) need(s) to be made. | Reported |
| Study perspective | 6 | Describe the perspective of the study and relate this to the costs being evaluated. | Reported (NHS) |
| Comparators | 7 | Describe the interventions or strategies being compared and state why they were chosen. | Reported |
| Time horizon | 8 | State the time horizon(s) over which costs and consequences are being evaluated and say why appropriate. | Reported (Lifetime) |
| Discount rate | 9 | Report the choice of discount rate(s) used for costs and outcomes and say why appropriate. |  |
|  | 9a | Discount rate for benefits | Reported (3%) |
|  | 9b | Discount rate for costs | Reported (3%) |
| Choice of health outcomes | 10 | Describe what outcomes were used as the measure(s) of benefit in the evaluation and their relevance for the type of analysis performed. | Reported |
|  | 10a | Form of economic evaluation  (declared by the authors) | Reported (cost-effectiveness) |
|  | 10b | Outcome measure | Reported (LYG  QALY) |
| Measurement of effectiveness | 11a | Number of sources of treatment  effects | Reported (1) |
|  | 11b | Sources of treatment effects in  single-study-based economic  evaluations | Reported (RCT) |
|  | 11c | Sources of treatment effects in  synthesis-based economic  evaluations | - |
| Measurement and valuation of preference based outcomes | 12 | If applicable, describe the population and methods used to elicit preferences for outcomes | - |
|  |  |  |  |
| Estimating resources and costs | 13a | Sources of resource consumption  in single-study-based economic  evaluations | Reported (data collection through questionnaire administration) |
|  | 13b | Sources of resource consumption  in synthesis-based economic  evaluations | - |
|  | 13c | Sources of monetary values to  estimate costs in single-study based  economic evaluations | Reported (official price/tariff list) |
|  | 13d | Sources of monetary values to  estimate costs in synthesis-based  economic evaluations | - |
|  | 13e | Type of costs | Reported (direct healthcare costs) |
| Currency, price date, and conversion | 14 | Report the dates of the estimated resource quantities and unit costs. Describe methods for adjusting estimated unit costs to the year of reported costs if necessary. Describe methods for converting costs into a common currency base and the exchange rate. |  |
|  | 14a | Currency | Reported (Eur) |
|  | 14b | Price date | Reported (2021) |
|  | 14c | Conversion | - |
| Choice of model | 15 | Describe and give reasons for the specific type of decision-analytical model used. Providing a figure to show model structure is strongly recommended. | Reported |
|  | 15a | Model design | Reported (partition survival model) |
|  | 15b | Discussion on choice of model | Reported |
|  | 15c | Figure of model structure | Reported |
| Assumptions | 16 | Describe all structural or other assumptions underpinning the decision-analytical model. | Reported |
| Analytical methods | 17 | Describe all analytical methods supporting the evaluation. This could include methods for dealing with skewed, missing, or censored data; extrapolation methods; methods for pooling data; approaches to validate or make adjustments (such as half cycle corrections) to a model; and methods for handling population heterogeneity and uncertainty. | Reported |
| **Results** | | | |
| Study parameters | 18 | Report the values, ranges, references, and, if used, probability distributions for all parameters. Report reasons or sources for distributions used to represent uncertainty where appropriate. Providing a table to show the input values is strongly recommended. | Reported |
| Incremental costs and outcomes | 19 | For each intervention, report mean values for the main categories of estimated costs and outcomes of interest, as well as mean differences between the comparator groups. If applicable, report incremental cost-effectiveness ratios. |  |
|  | 19a | Incremental costs | Reported |
|  | 19b | Incremental effectiveness | Reported |
|  | 19c | ICER | Reported |
|  | 19d | WTP threshold | Reported |
|  | 19e | Cost-effectiveness acceptability curves | Reported |
|  | 19f | Final recommendation | Reported |
|  | 19g | Policy recommendation | Reported |
|  | 19h | Technical conclusion | Reported (cost-effective) |
| Characterizing uncertainty | 20a | Uncertainty in single-study-based  economic evaluations | Reported (one-way  sensitivity analysis,  probabilistic sensitivity analysis) |
|  | 20b | Uncertainty in synthesis-based  economic evaluations | - |
| Characterizing heterogeneity | 21 | If applicable, report differences in costs, outcomes, or cost-effectiveness that can be explained by variations between subgroups of patients with different baseline characteristics or other observed variability in effects that are not reducible by more information. | Not reported |
| **Discussion** | | | |
| Study findings, limitations, generalizability, and current knowledge | 22 | Summarize key study findings and describe how they support the conclusions reached. Discuss limitations and the generalizability of the findings and how the findings fit with current knowledge. | Reported |
| **Other** | | | |
| Source of funding | 23 | Describe how the study was funded and the role of the funder in the identification, design, conduct, and reporting of the analysis. Describe other non-monetary sources of support. | Reported |
| Conflicts of interest | 24 | Describe any potential for conflict of interest of study contributors in accordance with journal policy. In the absence of a journal policy, we recommend authors comply with International Committee of Medical Journal Editors recommendations. | Reported |
| **Medical devices’ distinctive features** | | | |
| Learning curve | 25 |  | Substantial* |
| Incremental innovation | 26 |  | Substantial* |
| Dynamic pricing | 27 |  | Substantial* |
| Organizational impact | 28 |  | Substantial* |

* only mentioned in the text
