# Supplementary material for: Cost-effectiveness analysis of personalised versus standard dosimetry for selective internal radiation therapy with TheraSphere in patients with hepatocellular carcinoma
Source: Front Oncol. 2022 Aug 29;12:920073. doi: 10.3389/fonc.2022.920073 (PMC9464985; doi:10.3389/fonc.2022.920073)
Supplement: Supplementary file 2 [file DataSheet_2.docx]

**APPENDIX 2**

**Curves fitting**

The methodology followed few steps:

i) Digitization of the survival data of the Kaplan-Meier curves published in the reference study using Engauge Digitizer software.

ii) Analysis of digitized data with R and STATA software using the algorithms respectively of Guyot and colleagues [Guyot 2012] and Wei and colleagues [Wei 2017] in order to obtain the "fittings" through the main functions (exponential, Gompertz, log-logistic, log-normal, Weibull); these algorithms allow to map the digitized curves to Kaplan-Meier data by finding numerical solutions to the inverse Kaplan-Meier equations using information on the number of events and the number of subjects at risk. For a more detailed discussion, refer to Diaby and colleagues [Diaby 2014].

iii) The most appropriate curves (OS, PFS) were selected for standard dosimetry treatment. Statistical validity was assessed through the Akaike information criterion (AIC) and the Bayesian information criterion (BIC) (the lowest, the better fit).


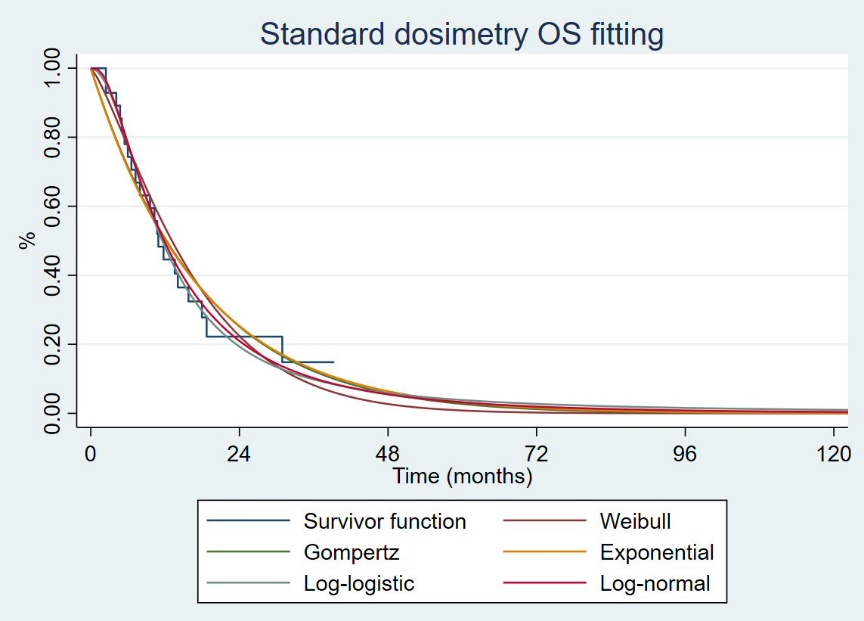


| **Standard dosimetry – OS curve** | | | |
| --- | --- | --- | --- |
| **Fitting** | **AIC** | **BIC** | **Notes** |
| Weibull | 74.62957 | 77.36416 |  |
| Gompertz | 76.40632 | 79.14091 |  |
| Exp | 74.41685 | 75.78415 |  |
| LogLog | 70.37439 | 73.10898 |  |
| LogN | **69.9537** | **72.68829** | Chosen fitting |


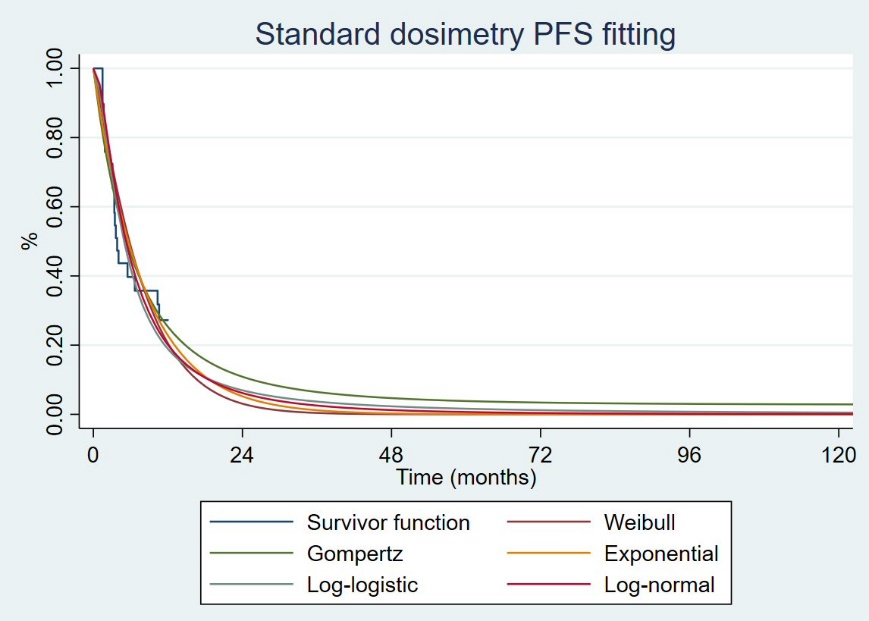


| **Standard dosimetry – PFS curve** | | | |
| --- | --- | --- | --- |
| **Fitting** | **AIC** | **BIC** | **Notes** |
| Weibull | 82.04782 | 84.78241 |  |
| Gompertz | 82.18478 | 84.91937 |  |
| Exp | 80.50588 | 81.87318 |  |
| LogLog | 77.84202 | 80.57661 |  |
| LogN | **76.4336** | **79.16819** | Chosen fitting |

**References**

Diaby V, Adunlin G, Montero AJ. Survival modeling for the estimation of transition probabilities in model-based economic evaluations in the absence of individual patient data: a tutorial. Pharmacoeconomics. 2014 Feb;32(2):101-8.

Guyot P, Ades AE, Ouwens MJ, Welton NJ. Enhanced secondary analysis of survival data: reconstructing the data from published Kaplan-Meier survival curves. BMC Med Res Methodol. 2012 Feb 1;12:9.

Wei Y, Royston P. Reconstructing time-to-event data from published Kaplan-Meier curves. Stata J. 2017 Oct;17(4):786-802.
