# Supplementary material for: Cost-effectiveness analysis of personalised versus standard dosimetry for selective internal radiation therapy with TheraSphere in patients with hepatocellular carcinoma
Source: Front Oncol. 2022 Aug 29;12:920073. doi: 10.3389/fonc.2022.920073 (PMC9464985; doi:10.3389/fonc.2022.920073)
Supplement: Supplementary file 3 [file Table_1.docx]

Supplementary Table 1 - Details of the parameters used in deterministic and probabilistic analyses

| **Name** | **Baseline value** | **Lower limit** | **Upper limit** | **Standard error of the mean** | **Distribution type** | **Alpha/mean of logs** | **Beta/lambda/standard error of logs** |
| --- | --- | --- | --- | --- | --- | --- | --- |
| Discount rate | 3% | 0.02 | 0.04 | 0.0 | Beta | 24.22 | 783.11 |
| *Population* |  |  |  |  |  |  |  |
| Age | 64.00 | 51.20 | 76.80 | 12.80 | Gamma | 25.00 | 2.56 |
| % males | 91.5% | 0.73 | 1.10 | 0.2 | Beta | 1.21 | 0.11 |
| *Hazard ratios (personalised vs. standard dosimetry)* |  |  |  |  |  |  |  |
| OS* | 0.421 | 0.215 | 0.826 | 0.156 | Log-normal | -0.865 | 0.469 |
| PFS* | 0.710 | 0.390 | 1.310 | 0.235 | Log-normal | -0.342 | 0.394 |
| *Health state utilities* |  |  |  |  |  |  |  |
| U Progression-free* | 0.51 | 0.42 | 0.60 | 0.046 | Beta | 59.59 | 57.25 |
| U Progression* | 0.35 | 0.25 | 0.45 | 0.050 | Beta | 31.50 | 58.50 |
| *Treatment costs* |  |  |  |  |  |  |  |
| Cost TARE simulation | € 4 085.00 | 3268.00 | 4902.00 | 817.00 | Gamma | 25.00 | 163.40 |
| Cost TARE | € 7 260.75 | 5808.60 | 8712.90 | 1452.15 | Gamma | 25.00 | 290.43 |
| Cost for personalised dosimetry | € 150.00 | 120.00 | 180.00 | 30.00 | Gamma | 25.00 | 6.00 |
| Cost liver resection | € 9 558.00 | 7646.40 | 11469.60 | 1911.60 | Gamma | 25.00 | 382.32 |
| Cost embolisation | € 4 085.00 | 3268.00 | 4902.00 | 817.00 | Gamma | 25.00 | 163.40 |
| Cost pharmacological treatments (PFS) | € 11.20 | 8.96 | 13.44 | 2.24 | Gamma | 25.00 | 0.45 |
| Cost pharmacological treatments (Progr.) | € 3.88 | 3.10 | 4.66 | 0.78 | Gamma | 25.00 | 0.16 |
| *Healthcare resource use frequencies (peri-procedural)* |  |  |  |  |  |  |  |
| Frequency specialist visit peri-procedural | 1.00 | 0.80 | 1.20 | 0.20 | Gamma | 25.00 | 0.04 |
| Frequency full blood counts peri-procedural | 1.00 | 0.80 | 1.20 | 0.20 | Gamma | 25.00 | 0.04 |
| Frequency creatinine peri-procedural | 1.00 | 0.80 | 1.20 | 0.20 | Gamma | 25.00 | 0.04 |
| Frequency sodium peri-procedural | 1.00 | 0.80 | 1.20 | 0.20 | Gamma | 25.00 | 0.04 |
| Frequency potassium peri-procedural | 1.00 | 0.80 | 1.20 | 0.20 | Gamma | 25.00 | 0.04 |
| Frequency calcium peri-procedural | 0.50 | 0.40 | 0.60 | 0.10 | Gamma | 25.00 | 0.02 |
| Frequency prothrombin time peri-procedural | 1.00 | 0.80 | 1.20 | 0.20 | Gamma | 25.00 | 0.04 |
| Frequency albumin peri-procedural | 1.00 | 0.80 | 1.20 | 0.20 | Gamma | 25.00 | 0.04 |
| Frequency bilirubin peri-procedural | 1.00 | 0.80 | 1.20 | 0.20 | Gamma | 25.00 | 0.04 |
| Frequency alpha-fetoprotein peri-procedural | 1.00 | 0.80 | 1.20 | 0.20 | Gamma | 25.00 | 0.04 |
| Frequency aspartate aminotransferase peri-procedural | 0.33 | 0.27 | 0.40 | 0.07 | Gamma | 25.00 | 0.01 |
| Frequency alanine aminotransferase peri-procedural | 1.00 | 0.80 | 1.20 | 0.20 | Gamma | 25.00 | 0.04 |
| Frequency gamma-glutamyl transpeptidase peri-procedural | 0.83 | 0.66 | 1.00 | 0.17 | Gamma | 25.00 | 0.03 |
| Frequency alkaline phosphatase peri-procedural | 1.00 | 0.80 | 1.20 | 0.20 | Gamma | 25.00 | 0.04 |
| Frequency pseudocholinesterase (PCHE) peri-procedural | 0.33 | 0.27 | 0.40 | 0.07 | Gamma | 25.00 | 0.01 |
| Frequency CT scan (abdomen) peri-procedural | 0.80 | 0.64 | 0.96 | 0.16 | Gamma | 25.00 | 0.03 |
| Frequency CT scan (torax) peri-procedural | 0.33 | 0.27 | 0.40 | 0.07 | Gamma | 25.00 | 0.01 |
| Frequency RMN abdomen peri-procedural | 0.20 | 0.16 | 0.24 | 0.04 | Gamma | 25.00 | 0.01 |
| Frequency ultrasound abdomen peri-procedural | 0.17 | 0.14 | 0.20 | 0.03 | Gamma | 25.00 | 0.01 |
| Frequency gastroscopy peri-procedural | 0.08 | 0.06 | 0.10 | 0.02 | Gamma | 25.00 | 0.00 |
| Frequency coil embolisation | 0.13 | 0.10 | 0.16 | 0.03 | Gamma | 25.00 | 0.01 |
| Frequency PET post treatment | 0.67 | 0.53 | 0.80 | 0.13 | Gamma | 25.00 | 0.03 |
| Frequency SPECT post simulation | 1.00 | 0.80 | 1.20 | 0.20 | Gamma | 25.00 | 0.04 |
|  |  |  |  |  |  |  |  |
| **PFS** |  |  |  |  |  |  |  |
| Frequency PFS specialist visit | 0.33 | 0.27 | 0.40 | 0.07 | Gamma | 25.00 | 0.01 |
| Frequency PFS full blood counts | 0.33 | 0.27 | 0.40 | 0.07 | Gamma | 25.00 | 0.01 |
| Frequency PFS creatinine | 0.33 | 0.27 | 0.40 | 0.07 | Gamma | 25.00 | 0.01 |
| Frequency PFS sodium | 0.33 | 0.27 | 0.40 | 0.07 | Gamma | 25.00 | 0.01 |
| Frequency PFS potassium | 0.33 | 0.27 | 0.40 | 0.07 | Gamma | 25.00 | 0.01 |
| Frequency PFS calcium | 0.17 | 0.13 | 0.20 | 0.03 | Gamma | 25.00 | 0.01 |
| Frequency PFS prothrombin time | 0.33 | 0.27 | 0.40 | 0.07 | Gamma | 25.00 | 0.01 |
| Frequency PFS albumin | 0.33 | 0.27 | 0.40 | 0.07 | Gamma | 25.00 | 0.01 |
| Frequency PFS bilirubin | 0.33 | 0.27 | 0.40 | 0.07 | Gamma | 25.00 | 0.01 |
| Frequency PFS alpha-fetoprotein | 0.33 | 0.27 | 0.40 | 0.07 | Gamma | 25.00 | 0.01 |
| Frequency PFS aspartate aminotransferase | 0.11 | 0.09 | 0.13 | 0.02 | Gamma | 25.00 | 0.00 |
| Frequency PFS alanine aminotransferase | 0.33 | 0.27 | 0.40 | 0.07 | Gamma | 25.00 | 0.01 |
| Frequency PFS gamma-glutamyl transpeptidase | 0.28 | 0.22 | 0.33 | 0.06 | Gamma | 25.00 | 0.01 |
| Frequency PFS alkaline phosphatase | 0.33 | 0.27 | 0.40 | 0.07 | Gamma | 25.00 | 0.01 |
| Frequency PFS pseudocholinesterase (PCHE) | 0.11 | 0.09 | 0.13 | 0.02 | Gamma | 25.00 | 0.00 |
| Frequency PFS CT scan (abdomen) | 0.27 | 0.21 | 0.32 | 0.05 | Gamma | 25.00 | 0.01 |
| Frequency PFS CT scan (torax) | 0.11 | 0.09 | 0.13 | 0.02 | Gamma | 25.00 | 0.00 |
| Frequency PFS RMN abdomen | 0.07 | 0.05 | 0.08 | 0.01 | Gamma | 25.00 | 0.00 |
| Frequency PFS ultrasound abdomen | 0.06 | 0.05 | 0.07 | 0.01 | Gamma | 25.00 | 0.00 |
| Frequency PFS gastroscopy | 0.03 | 0.02 | 0.03 | 0.01 | Gamma | 25.00 | 0.00 |
| **Progression** |  |  |  |  |  |  |  |
| Frequency Progression specialist visit | 1.00 | 0.80 | 1.20 | 0.20 | Gamma | 25.00 | 0.04 |
| Frequency Progression full blood counts | 1.00 | 0.80 | 1.20 | 0.20 | Gamma | 25.00 | 0.04 |
| Frequency Progression creatinine | 1.00 | 0.80 | 1.20 | 0.20 | Gamma | 25.00 | 0.04 |
| Frequency Progression sodium | 1.00 | 0.80 | 1.20 | 0.20 | Gamma | 25.00 | 0.04 |
| Frequency Progression potassium | 1.00 | 0.80 | 1.20 | 0.20 | Gamma | 25.00 | 0.04 |
| Frequency Progression calcium | 1.00 | 0.80 | 1.20 | 0.20 | Gamma | 25.00 | 0.04 |
| Frequency Progression prothrombin time | 1.00 | 0.80 | 1.20 | 0.20 | Gamma | 25.00 | 0.04 |
| Frequency Progression albumin | 1.00 | 0.80 | 1.20 | 0.20 | Gamma | 25.00 | 0.04 |
| Frequency Progression bilirubin | 1.00 | 0.80 | 1.20 | 0.20 | Gamma | 25.00 | 0.04 |
| Frequency Progression alpha-fetoprotein | 1.00 | 0.80 | 1.20 | 0.20 | Gamma | 25.00 | 0.04 |
| Frequency Progression alanine aminotransferase | 1.00 | 0.80 | 1.20 | 0.20 | Gamma | 25.00 | 0.04 |
| Frequency Progression gamma-glutamyl transpeptidase | 1.00 | 0.80 | 1.20 | 0.20 | Gamma | 25.00 | 0.04 |
| Frequency Progression alkaline phosphatase | 0.67 | 0.53 | 0.80 | 0.13 | Gamma | 25.00 | 0.03 |
| Frequency Progression CT scan (abdomen) | 0.16 | 0.12 | 0.19 | 0.03 | Gamma | 25.00 | 0.01 |
| Frequency Progression CT scan (torax) | 0.11 | 0.09 | 0.13 | 0.02 | Gamma | 25.00 | 0.00 |
| Frequency Progression RMN (abdomen) | 0.03 | 0.03 | 0.04 | 0.01 | Gamma | 25.00 | 0.00 |
| Frequency Progression ultrasound abdomen | 0.17 | 0.14 | 0.20 | 0.03 | Gamma | 25.00 | 0.01 |
| Frequency Progression paracentesis | 0.15 | 0.12 | 0.18 | 0.03 | Gamma | 25.00 | 0.01 |
| Frequency Progression gastroscopy | 0.01 | 0.01 | 0.01 | 0.00 | Gamma | 25.00 | 0.00 |
| *Healthcare resources costs* |  |  |  |  |  |  |  |
| Cost Specialist visit | € 20.66 | 16.53 | 24.79 | 4.13 | Gamma | 25.00 | 0.83 |
| Cost full blood counts | € 3.17 | 2.54 | 3.80 | 0.63 | Gamma | 25.00 | 0.13 |
| Cost creatinine | € 1.13 | 0.90 | 1.36 | 0.23 | Gamma | 25.00 | 0.05 |
| Cost sodium | € 1.02 | 0.82 | 1.22 | 0.20 | Gamma | 25.00 | 0.04 |
| Cost potassium | € 1.02 | 0.82 | 1.22 | 0.20 | Gamma | 25.00 | 0.04 |
| Cost calcium | € 1.13 | 0.90 | 1.36 | 0.23 | Gamma | 25.00 | 0.05 |
| Cost prothrombin time | € 2.85 | 2.28 | 3.42 | 0.57 | Gamma | 25.00 | 0.11 |
| Cost albumin | € 1.42 | 1.14 | 1.70 | 0.28 | Gamma | 25.00 | 0.06 |
| Cost bilirubin | € 1.13 | 0.90 | 1.36 | 0.23 | Gamma | 25.00 | 0.05 |
| Cost alpha-fetoprotein | € 7.40 | 5.92 | 8.88 | 1.48 | Gamma | 25.00 | 0.30 |
| Cost aspartate aminotransferase | € 1.04 | 0.83 | 1.25 | 0.21 | Gamma | 25.00 | 0.04 |
| Cost alanine aminotransferase | € 1.00 | 0.80 | 1.20 | 0.20 | Gamma | 25.00 | 0.04 |
| Cost gamma-glutamyl transpeptidase | € 1.13 | 0.90 | 1.36 | 0.23 | Gamma | 25.00 | 0.05 |
| Cost alkaline phosphatase | € 1.04 | 0.83 | 1.25 | 0.21 | Gamma | 25.00 | 0.04 |
| Cost pseudocholinesterase (PCHE) | € 1.36 | 1.09 | 1.63 | 0.27 | Gamma | 25.00 | 0.05 |
| Cost CT scan (abdomen) | € 103.68 | 82.94 | 124.42 | 20.74 | Gamma | 25.00 | 4.15 |
| Cost CT scan (torax) | € 77.67 | 62.14 | 93.20 | 15.53 | Gamma | 25.00 | 3.11 |
| Cost PET post treatment | € 1 071.65 | 857.32 | 1285.98 | 214.33 | Gamma | 25.00 | 42.87 |
| Cost SPECT post simulation | € 25.93 | 20.74 | 31.12 | 5.19 | Gamma | 25.00 | 1.04 |
| Cost RMN abdomen | € 120.08 | 96.06 | 144.09 | 24.02 | Gamma | 25.00 | 4.80 |
| Cost ultrasound abdomen | € 60.43 | 48.34 | 72.52 | 12.09 | Gamma | 25.00 | 2.42 |
| Cost paracentesis | € 34.86 | 27.89 | 41.83 | 6.97 | Gamma | 25.00 | 1.39 |
| Cost gastroscopy | € 56.81 | 45.45 | 68.17 | 11.36 | Gamma | 25.00 | 2.27 |
| Cost terminal care | € 4 374.00 | 3499.20 | 5248.80 | 874.80 | Gamma | 25.00 | 174.96 |
| *Adverse events frequencies* |  |  |  |  |  |  |  |
| **Personalised dosimetry** |  |  |  |  |  |  |  |
| Frequency lymphopenia | 34% | 0.27 | 0.41 | 0.07 | Gamma | 25.00 | 0.01 |
| Frequency increased aspartate aminotransferase | 9% | 0.07 | 0.10 | 0.02 | Gamma | 25.00 | 0.00 |
| Frequency anaemia | 6% | 0.05 | 0.07 | 0.01 | Gamma | 25.00 | 0.00 |
| Frequency increased alanine aminotransferase | 9% | 0.07 | 0.10 | 0.02 | Gamma | 25.00 | 0.00 |
| **Standard dosimetry** |  |  |  |  |  |  |  |
| Frequency lymphopenia | 43% | 0.34 | 0.51 | 0.09 | Gamma | 25.00 | 0.02 |
| Frequency asthenia | 5% | 0.04 | 0.06 | 0.01 | Gamma | 25.00 | 0.00 |
| Frequency ascites | 10% | 0.08 | 0.11 | 0.02 | Gamma | 25.00 | 0.00 |
| Frequency increased blood bilirubin | 5% | 0.04 | 0.06 | 0.01 | Gamma | 25.00 | 0.00 |
| Frequency increased aspartate aminotransferase | 10% | 0.08 | 0.11 | 0.02 | Gamma | 25.00 | 0.00 |
| Frequency anaemia | 5% | 0.04 | 0.06 | 0.01 | Gamma | 25.00 | 0.00 |
| Frequency thrombocytopenia | 5% | 0.04 | 0.06 | 0.01 | Gamma | 25.00 | 0.00 |
| Frequency decreased weight | 5% | 0.04 | 0.06 | 0.01 | Gamma | 25.00 | 0.00 |
| Frequency gastrointestinal haemorrhage | 10% | 0.08 | 0.11 | 0.02 | Gamma | 25.00 | 0.00 |
| Frequency icterus | 10% | 0.08 | 0.11 | 0.02 | Gamma | 25.00 | 0.00 |
| *Adverse events durations (days)* |  |  |  |  |  |  |  |
| Duration lymphopenia | 17 | 13.60 | 20.40 | 3.40 | Gamma | 25.00 | 0.68 |
| Duration asthenia | 17 | 13.60 | 20.40 | 3.40 | Gamma | 25.00 | 0.68 |
| Duration ascites | 23 | 18.40 | 27.60 | 4.60 | Gamma | 25.00 | 0.92 |
| Duration increased blood bilirubin | 21 | 16.80 | 25.20 | 4.20 | Gamma | 25.00 | 0.84 |
| Duration increased aspartate aminotransferase | 21 | 16.80 | 25.20 | 4.20 | Gamma | 25.00 | 0.84 |
| Duration anaemia | 23 | 18.40 | 27.60 | 4.60 | Gamma | 25.00 | 0.92 |
| Duration thrombocytopenia | 20 | 16.00 | 24.00 | 4.00 | Gamma | 25.00 | 0.80 |
| Duration decreased weight | 21 | 16.80 | 25.20 | 4.20 | Gamma | 25.00 | 0.84 |
| Duration increased alanine aminotransferase | 21 | 16.80 | 25.20 | 4.20 | Gamma | 25.00 | 0.84 |
| Duration gastrointestinal haemorrhage | 13 | 10.40 | 15.60 | 2.60 | Gamma | 25.00 | 0.52 |
| Duration icterus | 21 | 16.80 | 25.20 | 4.20 | Gamma | 25.00 | 0.84 |
| *Disutilities for adverse events* |  |  |  |  |  |  |  |
| Disutility lymphopenia* | 0.09 | 0.06 | 0.12 | 0.015 | Beta | 31.37 | 317.21 |
| Disutility asthenia* | 0.12 | 0.03 | 0.21 | 0.046 | Beta | 5.69 | 42.50 |
| Disutility ascites | 0.21 | 0.17 | 0.25 | 0.030 | Beta | 38.50 | 144.83 |
| Disutility increased blood bilirubin | 0.06 | 0.05 | 0.07 | 0.012 | Beta | 23.41 | 360.42 |
| Disutility increased aspartate aminotransferase | 0.06 | 0.05 | 0.07 | 0.012 | Beta | 23.41 | 360.42 |
| Disutility anaemia* | 0.11 | 0.07 | 0.15 | 0.022 | Beta | 21.18 | 171.40 |
| Disutility thrombocytopenia* | 0.23 | 0.02 | 0.44 | 0.106 | Beta | 3.39 | 11.36 |
| Disutility decreased weight | 0.03 | 0.02 | 0.03 | 0.006 | Beta | 24.25 | 811.82 |
| Disutility increased alanine aminotransferase | 0.06 | 0.05 | 0.07 | 0.012 | Beta | 23.41 | 360.42 |
| Disutility gastrointestinal haemorrhage | 0.11 | 0.09 | 0.13 | 0.021 | Beta | 22.22 | 185.43 |
| Disutility icterus | 0.30 | 0.24 | 0.36 | 0.060 | Beta | 17.20 | 40.13 |
| *Costs for the management of adverse events* |  |  |  |  |  |  |  |
| Cost lymphopenia | € 1 704 | 1363.20 | 2044.80 | 340.80 | Gamma | 25.00 | 68.16 |
| Cost asthenia | € 1 745 | 1396.00 | 2094.00 | 349.00 | Gamma | 25.00 | 69.80 |
| Cost ascites | € 1 748 | 1398.40 | 2097.60 | 349.60 | Gamma | 25.00 | 69.92 |
| Cost increased blood bilirubin | € 1 407 | 1125.60 | 1688.40 | 281.40 | Gamma | 25.00 | 56.28 |
| Cost increased aspartate aminotransferase | € 1 407 | 1125.60 | 1688.40 | 281.40 | Gamma | 25.00 | 56.28 |
| Cost anaemia | € 1 676 | 1340.80 | 2011.20 | 335.20 | Gamma | 25.00 | 67.04 |
| Cost thrombocytopenia | € 2 748 | 2198.40 | 3297.60 | 549.60 | Gamma | 25.00 | 109.92 |
| Cost decreased weight | € 1 758 | 1406.40 | 2109.60 | 351.60 | Gamma | 25.00 | 70.32 |
| Cost increased alanine aminotransferase | € 1 407 | 1125.60 | 1688.40 | 281.40 | Gamma | 25.00 | 56.28 |
| Cost gastrointestinal haemorrhage | € 959 | 767.20 | 1150.80 | 191.80 | Gamma | 25.00 | 38.36 |
| Cost icterus | € 1 407 | 1125.60 | 1688.40 | 281.40 | Gamma | 25.00 | 56.28 |

* variations according to 95%CI
